# Phosphine Oxide Containing Poly(pyridinium salt)s as Fire Retardant Materials

**DOI:** 10.3390/polym11071141

**Published:** 2019-07-03

**Authors:** Maksudul M. Alam, Bidyut Biswas, Alexi K. Nedeltchev, Haesook Han, Asanga D. Ranasinghe, Pradip K. Bhowmik, Kisholoy Goswami

**Affiliations:** 1 InnoSense LLC, 2531 West 237th Street, Torrance, CA 90505, USA; 2 Department of Chemistry and Biochemistry, University of Nevada Las Vegas, 4505 Maryland Parkway Box 454003, Las Vegas, NV 89154-4003, USA

**Keywords:** phosphine oxide, poly(pyridinium salt)s, fire retardant polymers, atomic force microscopy, luminescence, UV-Vis spectroscopy, microcalorimetry

## Abstract

Six new rugged, high-temperature tolerant phosphine oxide-containing poly(4,4′-(*p*-phenylene)-*bis*(2,6-diphenylpyridinium)) polymers **P-1**, **P-2**, **P-3**, **P-4**, **P-5**, and **P-6** are synthesized, characterized, and evaluated. Synthesis results in high yield and purity, as confirmed by elemental, proton (^1^H), and carbon 13 (^13^C) nuclear magnetic resonance (NMR) spectra analyses. High glass transition temperatures (**T*_g_* > 230 °C) and high char yields (>50% at 700 °C) are determined by differential scanning calorimetry (DSC) and thermogravimetric analysis (TGA), respectively. These new ionic polymers exhibit excellent processability, thin-film forming, high-temperature resistance, fire-resistance and retardation, coating, adhesion, mechanical and tensile strength, and n-type (electron transport) properties. The incorporation of phosphine oxide and bis(phenylpyridinium) moieties in the polymer backbones leads to high glass transition temperatures and excellent fire retardant properties, as determined by microcalorimetry measurements. The use of organic counterions allows these ionic polymers to be easily processable from several common organic solvents. A large variety of these polymers can be synthesized by utilizing structural variants of the bispyrylium salt, phosphine oxide containing diamine, and the counterion in a combinatorial fashion. These results make them very attractive for a number of applications, including as coating and structural component materials for automobiles, aircrafts, power and propulsion systems, firefighter garments, printed circuit boards, cabinets and housings for electronic and electrical components, construction materials, mattresses, carpets, upholstery and furniture, and paper-thin coatings for protecting important paper documents.

## 1. Introduction

Polymers are extensively used in our everyday lives due to their tunable properties and ease of processing. These qualities render polymers useful for applications ranging from adhesives and lubricants to structural components and windows for aircrafts. However, common polymers are also highly combustible, and produce toxic gases and smoke during combustion. Thus, the development of polymers with fire retardant properties is a major challenge. In recent years, regulations have required significant improvement of the fire performance of materials used in construction, transportation, and clothing. At the same time, some fire resistant materials containing bromine or chlorine moieties are being phased out due to their detrimental effects on health and the environment during combustion. Thus, the development of effective fire resistant polymers that can be produced and utilized safely and in an environmentally conscious manner is of great interest [1,2,3,4,5,6,7,8,9,10,11].

Over the past few years, nitrogen-containing polymers have received unabated attention in the design and synthesis of ionic polymers that have desirable electroluminescent (EL) [12,13,14,15,16,17,18,19,20,21,22,23,24,25], conducting [26], and liquid-crystalline (LC) properties [27,28,29,30,31,32,33,34,35,36,37,38,39,40,41], which make them attractive materials in many technological applications. Among the nitrogen-containing EL polymers, poly(pyridinium salt)s have received considerable interest and attention for applications in electronic and optoelectronic devices for displays and lighting, solar light harvesting, sensors, photonic devices, automobile and aircraft parts, and for packaging in the electronic industry. These ionic EL polymers exhibit many interesting properties, including redox behavior, electrical conductivity, electrochromism, photochromism, thermochromism, and LC properties [42,43,44,45,46,47,48,49,50,51,52,53,54,55,56,57,58]. The ionic polymers are also the appropriate polymers for the construction of functional multilayer assemblies by a sequential layer-by-layer deposition technique through electrostatic interactions [59,60,61,62,63,64,65,66]. The ionic EL polymers, which are also known as electroactive polymers (EAPs), render the ability to induce strains that are as high as two orders of magnitude greater than the movements that are possible with rigid and fragile electroactive ceramics [67]. EAP materials also have a higher response speed, lower density, and improved resilience when compared to shape memory alloys [67]. The synthesis of this class of photoactive and electroactive ionic polymers by the ring-transmutation polymerization reaction, and the characterization of their properties have been reported by several groups [42,43,44,45,46,47,48,49,50,51,52,53,54,55,56,57,58]. These ionic polymers have a high glass transition temperature, *T*_g_, and are thermally and thermo-oxidatively stable. They are highly crystalline and have excellent film-forming properties. Although poly(pyridinium) salts have very attractive properties and are thermally stable, they lack the robust, high-temperature, high-performance, high mechanical-strength, low-cost, and lightweight properties of ionic EL polymer materials.

Over the course of recent decades, conventional materials such as metals, wood, glass, and ceramic have been increasingly replaced by synthetic polymers due to their versatility, low density, mechanical properties, and the ease with which they can be molded. However, these advantages are shadowed by their flammability and low stability in high temperatures in comparison to metals. In recent years, considerable attention has been focused on preparing flame-retardant polymers; among these, phosphorus-containing polymers are the most widely used [1,2,3,4,5,6,7,8,9,10,11,68,69,70]. Phosphorus moieties have been incorporated into different polymeric backbones, including epoxy resin, poly(amic acid), polycarbonate, poly(vinyl chloride), polyester, polyimide, and poly(methyl methacrylate) [71,72,73,74,75,76,77,78]. Among the polymers with phosphorus-containing moieties, the polymers with phosphine oxide moieties have improved flame-retardant properties, thermal oxidative stability, solubility in organic solvents, miscibility, and adhesion to other compounds [79,80,81,82,83]. Although the incorporation of phosphine oxide moieties in the polymer backbone results in improved properties [84,85,86,87,88,89,90,91,92], the manufacturing costs with these polymers are prohibitive, because they are difficult to process. Currently, there is no robust high-temperature, high-performance, and lightweight phosphine oxide-containing electroactive and photoactive ionic polymer that can be manufactured and processed cost-effectively, and whose properties can be fine-tuned using easy synthetic routes. Thus, the development of easily processable and phosphine oxide-containing ionic EL polymers that have high temperature resistance and photoluminescence efficiency is highly desirable. The combination of ionic polymeric materials with a high-temperature resistant phosphine oxide group in the form of macromolecular architecture has the potential to deliver such a novelty.

**Figure 1:**
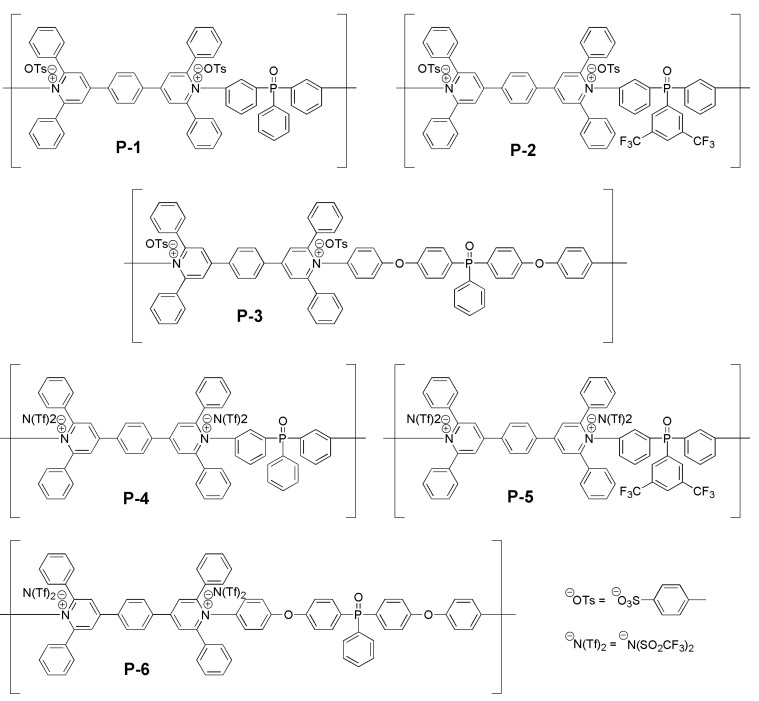
Chemical structures of rugged high temperature tolerant phosphine oxide-containing poly(4,4′-(*p*-phenylene)-*bis*(2,6-diphenylpyridinium)) polymers **P-1** to **P-6**.

In this study, we report on the design, synthesis, characterization, and evaluation of properties and the performance of six new phosphine oxide-containing poly(4,4′-(*p*-phenylene)-*bis*(2,6-diphenylpyridinium)) ionic polymers—**P-1**, **P-2**, **P-3**, **P-4**, **P-5**, and **P-6** (Figure 1)—using the ring-transmutation polymerization reaction. The thermal stability, fire-resistant, char yields, mechanical strength, coating and film forming, optical absorption, photoluminescence, electrochemical, and morphological properties of these ionic polymers have been studied to establish them as potential high-temperature and high-performance materials. The synthetic approach is versatile and amenable to low-cost mass production. This versatility stems from the ability of a large variety of these polymers to be synthesized by utilizing structural variants of the bispyridinium salt, phosphorous oxide-containing diamine, and the counterion in a combinatorial fashion. Thus, the chemical structural modifications allow the fine-tuning of their fire retardant properties. The purpose for using both phenylated bispyridinium ditosylate and phenyl phosphine oxide moieties in the backbone is to develop advanced polymeric structural materials with high thermal stability, high glass transition temperature, and enhanced photophysical, electrochemical, thermal, and mechanical properties.

**Figure 2:**
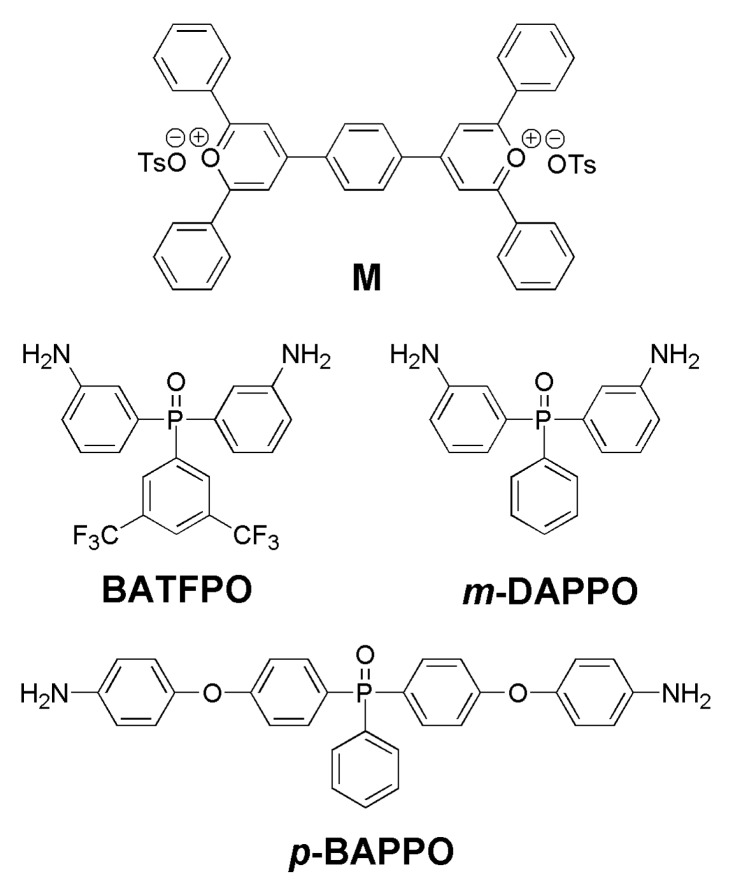
Chemical structures of 4,4′-(1,4-phenylene)-bis(2,6-diphenylpyrylium) ditosylate monomer (**M**), and phosphine oxide containing diamines, bis(3-aminophenyl)phenyl phosphine oxide (**m-DAPPO**), bis(3-aminophenyl)-3,5-bis(trifluoromethyl)phenyl phosphine oxide (**BATFPO**), and bis(4-aminophenoxy-4-phenyl) phenyl phosphine oxide (**p-BAPPO**).

The bispyrylium ditosylate monomer (**M** in Figure 2) is an excellent building block for the design and development of ionic polymers with both liquid-crystalline (LC) and light-emitting properties [42,43,44,45,46,47,48,49,50,51,52,53,54,55]. It contains a stable phenyl group and heteroatom in the aromatic ring that increase the thermal stability. Since these ionic polymers are cationic, they have great potential for building up multilayer assemblies with anionic polymers by sequential electrostatic deposition technique. The phenyl phosphine oxide containing aromatic diamines (bis(3-aminophenyl)phenyl phosphine oxide (**m-DAPPO**), bis(3-aminophenyl)-3,5-bis(trifluoromethyl)phenyl phosphine oxide (**BATFPO**), and bis(4-aminophenoxy-4-phenyl) phenyl phosphine oxide (**p-BAPPO**) as shown in Figure 2), were selected because of their high thermo-oxidative stability, high glass transition temperature, and the potential fire retardant behavior of phosphorous-containing polymers. The initial exposure of such polymers to atomic oxygen is expected to result in a passivating phosphate glassy layer on the surface, preventing further the erosion/corrosion caused by atomic oxygen in a harsh thermal environment [78,79,80,81,82,83,93,94,95].

## 2. Materialsand Methods

Terephthalaldehyde, acetophenone, triphenylmethanol, *p*-toluenesulfonic acid monohydrate, 3,5-*bis*(trifluoromethyl)bromobenzene, diphylphosphinic chloride, 4-aminophenol, phenylphosphonic dichloride, acetic acid, acetic anhydride, *p*-bromofluorobenzene, ethyl acetate, triphenyl phosphine oxide, nitric acid, sulfuric acid, hydrochloric acid, potassium hydroxide, sodium bicarbonate, Na_2_SO_4_, SnCl_2_·2H_2_O, Pd/C, and Mg turnings were purchased from TCI America (Portland, OR, USA) and used as received. For synthesis and purification purposes, reagent grade solvents including ethanol, propanol, toluene, benzene, chloroform, diethyl ether, hexane, tetrahydrofuran (THF), dichloromethane, ethylenechloride, acetonitrile, dimethyl sulfoxide (DMSO), and *N*,*N*-dimethylacetamide (DMAc) were used as obtained from Sigma-Aldrich (Milwaukee, WI, USA). Spectrophotometric grade solvents obtained from Sigma-Aldrich were used for optical absorption and fluorescence measurements. High-purity acetonitrile (purity >99.9%) obtained from Sigma-Aldrich was used for electrochemical measurements.

### 2.1. General Characterization Methods

The (proton) ^1^H and (carbon 13) ^13^C (nuclear magnetic resonance) NMR spectra of monomers and polymers were recorded on a Varian NMR J 400 spectrometer, 400 MHz at 298 K using both CDCl_3_ and *d*_6_-DMSO as solvents. Their elemental analyses were performed from Numega Resonance Laboratory, CA. To assess the molecular weight of the polymer, gel permeation chromatography (GPC) was run at 50 °C with a flow rate of 1 mL/min. The GPC instrument had a Water 515 pump simultaneously with a Viscotek Model 301 Triple Detector Array. The array contained a laser refractometer, a differential viscometer, and a light scattering detector both right angle laser light scattering (RALS) and low angle laser light scattering (LALS) in a single instrument with a fixed interdetector system and temperature control that can be regulated up to 80 °C. The instrument was calibrated with a pullulan standard of P-50 obtained from Polymer Standard Services USA, Inc. Separations were accomplished using ViscoGel I-MBHMW-3078 columns purchased from Viscotek. An aliquot of 100 to 200 mL of 2 mg/mL polymer solution in DMSO containing 0.01 M of LiBr was injected. The dn/dc (refractive index increment) values were corrected by injecting different volumes to assess the trend. All the data analyses were performed by using Viscotek TriSEC software. Thermogravimetric analysis of the polymer was conducted with a Universal V3.0G TA Instruments. A heating rate of 10 °C/min in N_2_ was used with runs being conducted from room temperature to 800 °C. Differential scanning calorimetry (DSC) measurements were run on a TA Instruments Q100 DSC in nitrogen. The heat–cool–heat method was used with an initial heating rate of 40 °C/min, a cooling rate of 20 °C/min, and a final heating rate of 10 °C/min in nitrogen. All the transitions were recorded from the second heating cycles of DSC thermograms. The optical absorption and fluorescence properties of these ionic polymers were studied using a Shimadzu UV-2401PC UV-Vis spectrophotometer and PTI QuantaMaster™ Model QM-4/2005 spectrofluorometer, respectively. Microcalorimetry measurements were done with a microscale combustion calorimeter (Govmark MCC-2) using 5 to 10-mg samples with a heating rate of 1 °C/sec to 750 °C in the pyrolysis zone. The combustion zone was set to 900 °C; oxygen and nitrogen flow rates were set at 20 mL/min and 80 mL/min, respectively. The microcalorimetry data are the average of three measurements on each sample.

### 2.2. Synthesis of 4,4’-(1,4-Phenylene)-bis(2,6-diphenylpyrylium)ditosylate Monomer (M)

**Scheme 1:**
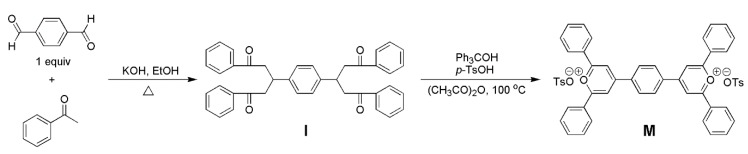
Synthesis of 4,4’-(1,4-phenylene)-*bis*(2,6-diphenylpyrylium) ditosylate monomer (**M**).

A mixture of terephthalaldehyde (10.0 g) and acetophenone (54.3 g) was stirred in 250 mL of 95% ethanol at 65 °C. After the starting compounds were dissolved, a solution of KOH (10.5 g) in 10 mL of water was added dropwise over 30 min with vigorous stirring. A yellow precipitate formed immediately. Then, the heterogeneous reaction mixture was heated at reflux until it turned pink over a period of 5 h. During this time, the *p*-bischalcone was redissolved and reacted with two additional equivalents of acetophenone to form the desired tetraketone, **I** (Scheme 1), which was also precipitated out. The reaction mixture was filtered hot, and the tan solid was collected by filtration to afford 41.0 g of the crude product. It was recrystallized from toluene to afford 38.0 g (yield: 89%) of off-white crystals of compound **I** (Scheme 1). The chemical structure and purity of **I** were confirmed by elemental and ^1^H NMR analyses [54].

For the conversion of **I** to **M** (the second step in Scheme 1), triphenylmethanol (7.8 g) and *p*-toluenesulfonic acid monohydrate (5.8 g) were added to 100 mL of acetic anhydride, and stirred at room temperature for 3 h. Then, solid tetraketone **I** (7.2 g) was added to the reaction mixture, and the mixture was heated to 100 °C for 1 h. The heterogeneous mixture became clear. Upon cooling, yellow crystals appeared and were collected by filtration, after which they were washed carefully with acetic anhydride and ethanol. Then, the product was recrystallized from acetic acid and dried in vacuum to afford 7.9 g of **M** (yield 75%) [54]. ^1^H NMR of **M** in *d*_6_-DMSO, d_H_: 9.35 (4H, s, aromatic meta O^+^), 9.21 (4H, s, 1,4-phenylene), 7.58–8.93 (20H, m, phenyl), 7.46–7.47 (4H, d, *J* = 6.7 Hz, tosylate), 7.09–7.10 (4H, d, *J* = 7.7 Hz, tosylate), 2.27 (6H, s, CH_3_).

### 2.3. Bis(3-aminophenyl)phenyl Phosphine Oxide (m-DAPPO)

An amount of 11.14 g of triphenyl phosphine oxide (**TPO**), **II**, was placed in a 250-mL flask equipped with a stirrer, nitrogen inlet, and a thermometer. A volume of 60 mL of 96% H_2_SO_4_ was added, and once the reactant was dissolved, the solution was cooled to −5 °C with an ice/salt bath. A solution of 5.80 g of 90% fuming HNO_3_ in 40 mL of H_2_SO_4_ was added dropwise over a period of 1 h. The samples were maintained at −5 °C during the addition, increased to room temperature (rt), and left at this temperature for 8 h. The obtained pale yellow solution was poured into 400 mL of ice water, resulting in a sticky solid that was collected by decantation. Then, this solid was dissolved in CHCl_3_ and washed with aqueous sodium bicarbonate solution until it was neutral. The organic layer was dried over Na_2_SO_4_ overnight and filtered. The solvent was removed by rotary evaporation. The crude product was dried in vacuo and recrystallized twice from ethanol to produce 9.6 g (26.1 mmol, yield 65%) of pure product [96,97]. Elemental and ^1^H NMR analyses were performed to confirm the chemical structure of **m-DNPPO**. In the second step, **m-DNPPO** (5.0 g) was added to a solution of SnCl_2_·2H_2_O in 50 mL of concentrated HCl and 100 mL of ethanol. This mixture was left stirring at rt for 2 h and then heated for an additional 2 h. The reaction mixture was cooled down to rt and poured in 60 g of KOH in 200 mL of ice water and stirred vigorously. The solid crude product was filtered, washed with H_2_O until neutral, and recrystallized from chloroform to give 3.5 g (11.4 mmol, yield 84%) [98]. The chemical structure of **m-DAPPO** was confirmed by elemental, ^1^H and ^13^C NMR spectra analyses. Anal. Calcd. for C_18_H_17_N_2_OP (308.32): C 70.12, H 5.56, N 9.09; found: C 70.17, H 5.82, N 9.43.

### 2.4. Bis(3-aminophenyl)-3,5-bis(trifluoromethyl)phenyl Phosphine Oxide (BATFPO)

An amount of 1.05 g of Mg turnings and 50 mL of ether were charged into a 250-mL three-necked, round-bottomed flask, equipped with magnetic stirrer, condenser, drying tube, thermometer, and nitrogen inlet. The solution was cooled to below 5 °C in an ice bath; then, 9.71 g of 3,5-*bis*(trifluoromethyl)bromobenzene was added dropwise over a period of 1 h, while stirring vigorously. Then, the mixture was allowed to react for an additional 5 h. Then, 7.00 g of diphylphosphinic chloride was added dropwise over a period of 1 h. After reacting for an additional 18 h, a brown solution was obtained. Next, 10% aqueous sulfuric acid was added to the solution to a pH of 1, followed by the addition of 250 mL of water and diethyl ether to form aqueous and organic layers. After decanting the ether layer, the aqueous phase was washed twice with diethyl ether, and all the organic layers were combined and dried by evaporation, resulting in 3,5-*bis*(trifluoromethyl)phenyl diphenyl phosphine oxide (**TFPO**), which was a light brown solid. Then, **TFPO** was dissolved in chloroform and washed several times with 10% sodium bicarbonate and three times with water. The organic layer was condensed by rotary evaporator and stored at room temperature for 12 h and another 12 h in a freezer. The fibrous off-white crystals of **TFPO** were collected by vacuum filtration and further purified by recrystallization in hexane [99].

In the second step, *bis*(3-nitrophenyl)-3,5-*bis*(trifluoromethyl)phenyl phosphine oxide (**DNTFPO**) was prepared by the nitration of **TFPO** using concentrated sulfuric and concentrated nitric acid. Purified **TFPO** (10.0 g) was added into a 250-mL three-necked flask equipped with a nitrogen inlet, thermometer, drying tube, and mechanical stirrer. Concentrated sulfuric acid (23 mL) was added into the flask to dissolve the compound **TFPO** at room temperature. The solution was cooled down to 0 °C with an ice-water bath. Nitric acid (4.7 mL) was added dropwise to the solution over a period of 1 h, while stirring vigorously and maintaining 0 °C. Then, the mixture was allowed to react for 8 h and poured into 650 g of finely divided ice. The resulting yellowish solid was extracted with chloroform, and then followed by washing with sodium bicarbonate aqueous solution until the pH reached 7. The solvent was removed with a rotary evaporator, and the remaining solid was recrystallized twice from absolute ethanol, which afforded 10.0 g of pale-yellow crystals of **DNTFPO [99]**.

Then, *bis*(3-aminophenyl)-3,5-*bis*(trifluoromethyl)phenyl phosphine oxide (**BATFPO**) was prepared by the reduction of **DNTFPO** by the stannous chloride method [30]. An amount of 4.0 g of BATFPO was added to a solution of 10.8 g of SnCl_2_·2H_2_O in 30 mL of concentrated HCl and 60 mL of ethanol, and left stirring at rt for 2 h and heated for an additional 2 h. The reaction mixture was cooled down to room temperature, poured into a solution of 40 g of KOH in 200 mL of ice water, and stirred vigorously. The solid crude product was filtered and washed with copious amount of H_2_O until neutral, giving the crude product of **BATFPO**, and it was air-dried. Then, it was further purified by sublimation to afford 3.0 g (yield 86%). The ^1^H and ^13^C NMR spectra, and elemental analysis confirmed its chemical structure and purity. Anal. Calcd. for C_20_H_15_N_2_OF_6_P (444.32): C 54.07, H 3.40, N 6.30; found: C 53.88, H 3.80, N 6.39. It showed a *T*_m_ at 228 °C with ΔH = 9.0 kcal/mol in the first heating cycle of the DSC thermogram (lit. mp = 226–227 °C) [99].

### 2.5. Bis(4-aminophenoxy-4-phenyl) Phenyl Phosphine Oxide (p-BAPPO)

First, a solution of *p*-bromofluorobenzene (15 g) in THF (40 mL) was added dropwise to a slurry of 2.2 g of magnesium in 50 mL of THF in an ice bath over a period of 3 h. This was allowed to stir overnight at room temperature. During this time, a gray-colored solution appeared. The mixture was again cooled to 0 °C, and a solution of phenylphosphonic dichloride (8.35 g) in 20 mL of THF was added dropwise over a period of 3-4 h with stirring. Then, the mixture was warmed to room temperature and stirred for 12 h. The mixture was quenched with 10% H_2_SO_4_ and stirred for 1 h. Ether (200 mL) was added to separate the organic layer, and the aqueous layer was extracted with ether (3×75 mL). The combined ethereal extract was washed with NaHCO_3_ solution and water, and dried over sodium sulfate. The solvent was removed under reduced pressure to furnish a light brown oil. The crude product was purified by crystallization from THF/hexane (1:1) to afford *bis*(4-fluorophenyl)-phenylphosphineoxide (***p*-FPPO**) as an off-white solid (89% yield), mp 128–130 °C. In the second step, a magnetically stirred mixture of ***p*-FPPO** (4 g), 4-aminophenol (3.06 g), and K_2_CO_3_ (5.28 g) in DMAc (15 mL) was heated to reflux for 24 h in nitrogen. Then, the reaction mixture was cooled to rt and poured into ice water with vigorous stirring. The precipitated off-white solid product was collected in a Buchner funnel using vacuum filtration, and washed well with water to remove the salt and unreacted 4-aminophenol. The crude product was finally purified by column chromatography over silica gel eluting with 2% methanol/98% ethyl acetate (v/v) to furnish ***p*-BAPPO** as an off-white solid powder with 82% yield. The chemical structure was confirmed by ^1^H and ^13^C NMR and DSC analysis (mp 98-100 °C) [100].

### 2.6. Phenylphosphine Oxide-Containing Poly(4,4′-(p-phenylene)-bis(2,6-diphenylpyridinium)) Ionic Polymers

The first phenylphosphine oxide-containing poly(4,4′-(*p*-phenylene)-*bis*(2,6-diphenylpyridinium)) ionic polymer (**P-1**) was prepared by the ring-transmutation polymerization reaction [42,43,44,45,46,47,48,49,50,51,52,53,54,55,56,57,58] as follows: 6.5873 g of **M** was polymerized with **m-DAPPO** (2.3000 g) on heating in DMSO at 130–140 °C for 24 h (Scheme 5). The water generated during the polymerization was distilled out from the reaction medium as a toluene/water azeotrope. The yellowish solid **P-1** polymer was isolated with 80% yield by two cycles of precipitation with distilled water and dissolution in methanol. **P-1** was fully characterized by elemental analysis, and ^1^H and ^13^C NMR spectra analyses. Anal. Calcd. for C_72_H_55_N_2_O_7_S_2_P (1155.33): C 74.85, H 4.80, N 2.42, S 5.55; found: C 73.36, H 4.82, N 2.44, S 5.45.

Using the identical ring-transmutation polymerization reaction (Scheme 5) and purification processes, an ionic polymer **P-2** (77% yield; yellow solid) was obtained by polymerizing **M** (8.4800 g, 6.56 mmol) with **BATFPO** (2.918 g, 6.56 mmol) in DMSO at 130-140 °C for 24 h. The polymer was essentially isolated in a quantitative yield by precipitation with distilled water. It was further purified by redissolving in methanol and by subsequent re-precipitation with the addition of distilled water. Similarly, an ionic polymer **P-3** (79% yield; dark brown solid) was obtained by polymerizing **M** (5.3787 g, 6.09 mmol) with ***p*-BAPPO** (3.0000 g, 6.09 mmol) in DMSO at 130–140 °C for 24 h. The polymer was isolated in a quantitative yield by precipitation with distilled water. It was further purified by redissolving in methanol and by subsequent re-precipitation with the addition of distilled water to yield ionic polymer **P-3**. The chemical structures of both ionic polymers **P-2** and **P-3** were also confirmed by elemental, ^1^H, and ^13^C NMR spectra analyses (see Supplementary Materials). Elemental analysis for **P-2** polymer, Anal. Calcd. for C_79_H_53_N_2_O_7_F_6_S_2_P (1291.34): C 68.83, H 4.14, N 2.17, S 4.97; found: C 66.38, H 4.69, N 2.28, S 5.74. Elemental analysis for **P-3** polymer, Anal. Calcd. for C_84_H_63_N_2_O_9_S_2_P (1339.55): C 75.32, H 4.74, N 2.09, S 4.79; found: C 71.87, H, 5.24, N 2.05, S 5.00.

Ionic polymer **P-4** was prepared by the metathesis reaction of ionic polymer **P-1** with lithium triflimide in DMSO (Scheme 6) [42,43,44,45,46,47,48,49,50,51,52,53,54,55] First, 1.40 g (1.21 mmol) of polymer **P-1** was dissolved in 50 mL of DMSO. To the DMSO solution of polymer **P-1**, lithium triflimide (0.73 g, 2.54 mmol) was slowly added. The resulting solution was kept at 50 °C for 48 h on continuous stirring. After reducing the volume of DMSO solution by a rotary evaporator, the reaction mixture was added to distilled water, affording the desired ionic polymer **P-4**. It was collected by vacuum filtration, washed several times with a large quantity of hot distilled water, and dried in vacuum at 100 °C for 72 h and weighed to give 1.58 g (1.50 mmol) of polymer **P-4** (yield 95%). Anal. Calcd. for C_62_H_41_N_4_O_9_F_12_S_4_P (1373.24): C 54.23, H 3.01, N 4.08, S 9.34; found: C 54.41, H 3.19, N 4.02, S 9.05.

Using a similar metathesis reaction, ionic polymer **P-5** was prepared from ionic polymer **P-2**. First, 1.40 g (1.08 mmol) of **P-2** was dissolved in 50 mL of DMSO. Then, lithium triflimide (0.73 g, 2.28 mmol) was added to the DMSO solution of this polymer. The resulting solution was kept at 50 °C for 48 h. After reducing the DMSO solution by a rotary evaporator, the reaction mixture was added to distilled water, affording the desired ionic polymer **P-5**. It was collected by vacuum filtration and washed several times with a large quantity of hot distilled water. This procedure was repeated once more; then, the collected polymer was dried in vacuum at 100 °C for 72 h and weighted to give 1.54 g (1.02 mmol) of polymer **P-5**. Unfortunately, the ^1^H NMR spectrum of this polymer showed a pair of doublets [δ = 7.09 (d) and 7.46 ppm (d)], suggesting the incomplete exchange of tosylate ions by triflimide ions. To complete the exchange of tosylate ions to produce polymer **P-5**, a metathesis reaction was carried out for the third time yielding the successful metathesis reaction. Using the identical procedure, ionic polymer **P-6** was prepared by the metathesis reaction of **P-3** with lithium triflimide in DMSO. The chemical structures and purities of ionic polymers **P-5** and **P-6** were confirmed by elemental, ^1^H, and ^13^C NMR spectra analyses (see Supplementary Materials). Elemental analysis for polymer **P-5**, Anal. Calcd. for C_64_H_39_N_4_O_9_F_18_S_4_P (1509.23): C 50.93, H 2.60, N 3.71, S 8.50; found: C 50.26, H 2.71, N 3.75, S 10.25. Elemental analysis for polymer **P-6**, Anal. Calcd. for C_74_H_49_N_4_O_11_F_12_S_4_P (1557.43): C 57.07, H 3.17, N 3.60, S 8.23; found: C 54.57, H 3.77, N 3.55, S 8.88.

### 2.7. Cyclic Voltammetry Measurements

Cyclic voltammetry experiments were done on an EG&G Princeton Applied Research potentiostat/galvanostat (model 263A). Data were collected and analyzed by the model 270 Electrochemical Analysis System software. A three-electrode electrochemical cell was used in all the experiments, as previously described [101,102]. Platinum wire electrodes were used as both counter and working electrodes, and silver/silver ion (Ag in 0.1 M AgNO_3_ solution, Bioanalytical System, Inc.) was used as a reference electrode. The Ag/Ag+ (AgNO_3_) reference electrode was calibrated at the beginning of the experiments by running cyclic voltammetry on ferrocene/ferrocenium ions as the internal standard. By means of the internal ferrocenium/ferrocene (Fc^+^/Fc) standard, the potential values, which were obtained in reference to the Ag/Ag+ electrode, were converted to the saturated calomel electrode (SCE) scale. The films of all the ionic polymers were coated on the Pt working electrode by dipping the Pt wire into the viscous solution in methanol, and then drying it in a vacuum oven at 80 °C for 8 h. An electrolyte solution of 0.1 M of TBAPF_6_ in a mixed water/acetonitrile solvent was used in all the experiments. All the solutions in the three-electrode cell were purged with ultrahigh-purity nitrogen for 10–15 min before each experiment, and a blanket of N_2_ was used during the experiment.

## 3. Results and Discussion

### 3.1. Synthesis and Characterization of Monomers

4,4′-(1,4-phenylene)-*bis*(2,6-diphenylpyrylium) ditosylate monomer (**M**) was synthesized according to the known procedures in two steps (Scheme 1) [54]. In the first step, terephthalaldehyde was condensed with acetophenone to afford the desired tetraketone, **I**. Then, the product was recrystallized from toluene to afford 38.0 g (yield: 89%) of off-white crystals of compound **I**. The chemical structure and purity of **I** were confirmed by elemental, ^1^H NMR, and differential scanning calorimetry (DSC) (melting endotherm at 206 °C) analyses. In the second step (Scheme 1), tetraketone **I** was subsequently cyclodehydrated to monomer **M** by treatment with triphenylmethyl tosylate, which is a hydride acceptor. This hydride acceptor was generated in situ from triphenylmethanol and tosic acid. The product was collected by filtration, washed carefully with (CH_3_CO)_2_O and ethanol, recrystallized from acetic acid, and finally obtained as yellow crystals of the desired 4,4′-(1,4-phenylene)-*bis*(2,6-diphenylpyrylium) ditosylate monomer with a yield of 75%. The chemical structure and purity of **M** were confirmed by ^1^H and ^13^C NMR and elemental analyses. The NMR peak positions and the integration ratio between the aromatic protons and the aliphatic protons are in excellent agreement with the calculated values of the compound. Several endotherms by DSC analyses were observed at 161 °C (*T*_m_), 195 °C, and 304 °C (T_i_), which match with the previously reported values [54].

**Scheme 2:**
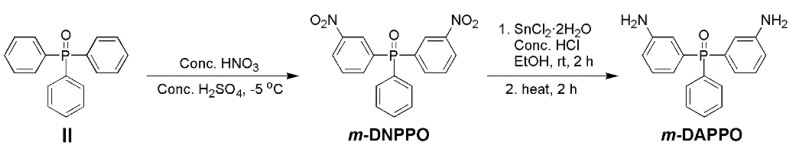
Synthesis of *bis*(3-aminophenyl) phenyl phosphine oxide (**m-DAPPO**).

*Bis*(3-aminophenyl)phenyl phosphine oxide (**m-DAPPO**) was synthesized by a two-step process (Scheme 2) [96,97]. In the first step, triphenyl phosphine oxide (**TPO**), **II**, was reacted with 90% fuming HNO_3_ and 96% H_2_SO_4_ to afford *bis*(3-nitrophenyl)phenyl phosphine oxide (**m-DNPPO**). The crude product was dried in vacuum and recrystallized twice from ethanol and obtained **m-DNPPO** (yield: 65%). Elemental and ^1^H NMR analyses confirmed the chemical structure and high purity of **m-DNPPO**. In the second step, *bis*(3-aminophenyl)phenyl phosphine oxide (**m-DAPPO**) was prepared by the reduction of **m-DNPPO** by stannous chloride method [98]. This method was used for the synthesis of this diamine, since the reduction with hydrogen over Pd/C in the hydrogenation shaker produced a low yield of 28% [96]. The solid crude product was filtered, washed with H_2_O until neutral, and recrystallized from chloroform to give **m-DAPPO** (yield: 84%). The chemical structure and high purity of **m-DAPPO** were also confirmed by elemental, ^1^H, and ^13^C NMR analyses. DSC thermograms of **m-DAPPO** were obtained at heating and cooling rates of 10 °C/min in nitrogen. In the first heating cycle, the peak maximum of the melting endotherm was 208 °C, which agrees well with the reported value of the melting point (mp = 203 °C) [96,97].

**Scheme 3:**
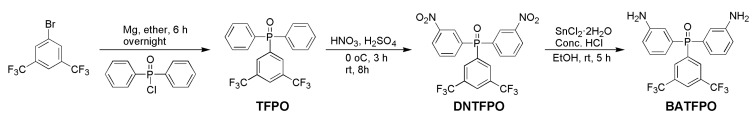
Synthesis of *bis*(3-aminophenyl)-3,5-bis(trifluoromethyl) phenyl phosphine oxide (**BATFPO**).

Scheme 3 outlines the synthesis of *bis*(3-aminophenyl)-3,5-*bis*(trifluoromethyl)phenyl phosphine oxide (**BATFPO**). This diamine was synthesized via the Grignard reaction prepared from 3,5-*bis*(trifluoromethyl)bromobenzene with diphenylphophinic chloride, followed by nitration and reduction reactions [98,99]. The solid crude product was filtered and washed with the copious amount of water until neutral, which gave a crude product of **BATFPO**, and was air-dried. Then, it was further purified by sublimation to afford pure **BATFPO** (yield 86%). ^1^H and ^13^C NMR spectra and elemental analyses confirmed its chemical structure and purity. It showed a *T*_m_ at 228 °C with ΔH = 9.0 kcal/mol in the first heating cycle of the DSC thermogram (lit. mp = 226–227 °C) [99].

**Scheme 4:**
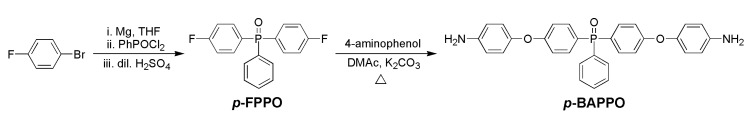
Synthesis of *bis*(4-aminophenoxy-4-phenyl) phenyl phosphine oxide (***p*-BAPPO**).

The third diamine compound *bis*(4-aminophenoxy-4-phenyl) phenyl phosphine oxide (***p*-BAPPO**) was synthesized in a two-step method (Scheme 4). First, *bis*(4-fluorophenyl)-phenylphosphineoxide (***p*-FPPO**) as an off-white solid with a yield of 89% and mp = 128–130 °C was prepared by reacting *p*-bromofluorobenzene with phenylphosphonic dichloride in the presence of magnesium in tetrahydrofuran (THF) [100]. In the second step, ***p*-FPPO** was reacted with 4-aminophenol in the presence of K_2_CO_3_ in *N*,*N*-dimethyl acetamide (DMAc) to afford ***p*-BAPPO**. The crude product was finally purified by column chromatography over silica gel eluting with 2% methanol/98% ethyl acetate (v/v) to furnish ***p*-BAPPO** as an off-white solid powder with 82% yield. The chemical structure was confirmed by ^1^H and ^13^C NMR and DSC analyses (mp = 98–100 °C) [100].

### 3.2. Synthesis and Characterization of Ionic Polymers

**Scheme 5:**
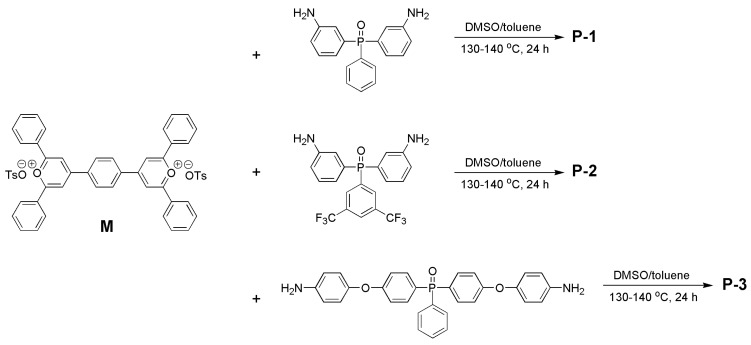
Synthesis of poly(4,4′-(*p*-phenylene)-*bis*(2,6-diphenylpyridinium)) polymer **P-1** using a ring-transmutation polymerization reaction.

**Figure 3:**
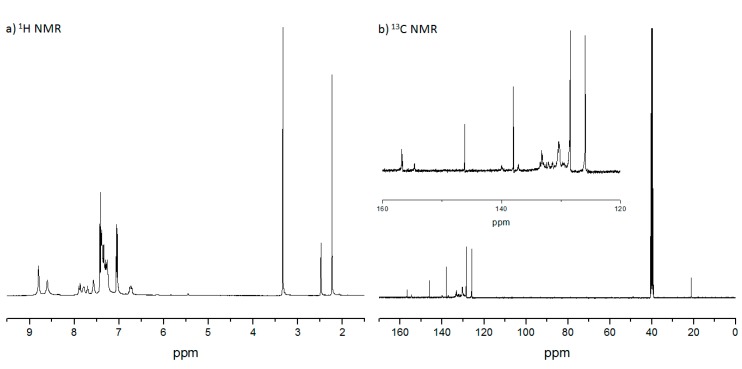
^1^H and ^13^C NMR spectra of ionic polymer **P-1** in *d*_6_-DMSO taken at room temperature.

**Figure 4:**
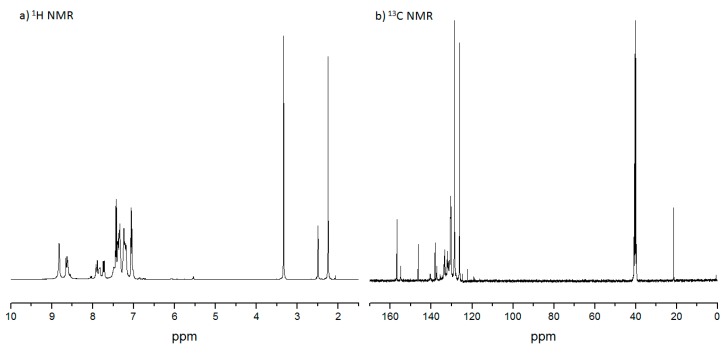
^1^H and ^13^C NMR spectra of ionic polymer **P-2** in *d*_6_DMSO taken at room temperature.

The phenyl phosphine oxide-containing poly(4,4′-(*p*-phenylene)-*bis*(2,6-diphenylpyridinium)) ionic polymer (**P-1**) was prepared by a ring-transmutation polymerization reaction (Scheme 5) [42,43,44,45,46,47,48,49,50,51,52,53,54,55,56,57,58]. This polymerization reaction was essentially a polycondensation reaction between 4,4′-(1,4-phenylene)-*bis*(2,6-diphenylpyrylium) ditosylate monomer (**M**) and *bis*(3-aminophenyl)phenyl phosphine oxide (**m-DAPPO**) liberating water as a condensation product. **M** was polymerized with **m-DAPPO** simply on heating in non-volatile, non-toxic, water-miscible solvent dimethyl sulfoxide (DMSO) at 130–140 °C for 24 h. Regarding the water, the condensation product that was generated during the polymerization reaction was distilled out from the reaction medium as a toluene/water azeotrope. The yellowish solid ionic polymer **P-1** was isolated by two cycles of precipitation with distilled water and dissolution in methanol. Its yield of 77–80% with high purity was achieved after purification. It should be noted that the reaction medium was maintained as a homogeneous solution throughout the entire polymerization reaction period, thus permitting the production of a high molecular weight polymer. This polycondensation reaction was essentially in contrast to other polycondensation reactions in which polymers usually precipitate out of solutions prematurely, thus limiting the maximum molecular weights of the polymers. Polymer **P-1** was fully characterized by elemental analysis, ^1^H and ^13^C NMR, and gel permeation chromatography (GPC) analyses. Figure 3 shows the ^1^H and ^13^C NMR spectra of ionic polymer **P-1** in *d*_6_-DMSO at room temperature. Its ^1^H NMR spectrum showed unique resonances at δ = 8.93 and 8.70 ppm for the protons of the aromatic moieties of bispyridinium salts and a set of resonances at δ = 7.44 and 7.05 ppm and 2.25 ppm for the protons of the aromatic moiety and methyl group in the tosylate counterion. The relative integration ratio of all the aromatic protons (49H) and the aliphatic protons (6H) is in excellent agreement with the calculated value obtained from those in the repeating unit of this polymer. Its ^13^C NMR spectrum contained both aliphatic and aromatic carbon signals at appropriate chemical shifts, as expected. Using similar ring-transmutation polymerization reaction (Scheme 5) and purification processes, ionic polymers **P-2** and **P-3** were obtained by polymerizing **M** with the corresponding diamines (**BATFPO** and ***p*-BAPPO**) in DMSO at 130–140 °C for 24 h with yields of 77% (yellow solid) and 79% (dark brown solid), respectively. The chemical structures of both the ionic polymers **P-2** and **P-3** were also confirmed by elemental, ^1^H, and ^13^C NMR spectra analyses. Figure 4 shows the ^1^H and ^13^C NMR spectra of polymer **P-2** in *d*_6_-DMSO taken at room temperature, and the ^1^H and ^13^C NMR spectra of polymer **P-3** are given in Figure S1. Similar to the analysis of ^1^H NMR spectrum of polymer **P-1** (*vide supra*), its relative integration ratio of the aromatic protons (47H) and aliphatic protons (6H) is in excellent agreement with the calculated value obtained from those in the repeating unit of this polymer. Its ^13^C NMR spectrum also contained both aliphatic and aromatic carbon signals at appropriate chemical shifts, as expected.

**Scheme 6:**
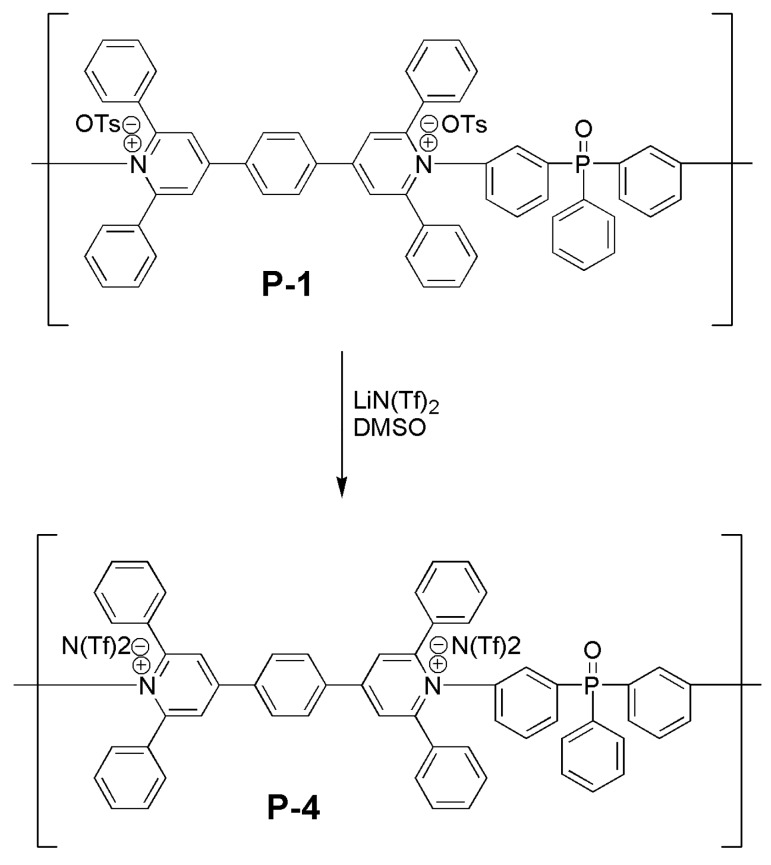
Metathesis reaction for the conversion of ionic polymer **P-1** to **P-4**.

Additional ionic polymers were prepared by a simple metathesis reaction of the respective primary ionic polymers in the presence of a desired ion (Scheme 6) [42,43,44,45,46,47,48,49,50,51,52,53,54,55]. This reaction leads to the exchange of the original tosylate ions with newly introduced ions. Using this metathesis reaction, ionic polymers **P-4**, **P-5**, and **P-6** were prepared with high yield (95%) and purity from the respective ionic polymers **P-1**, **P-2**, and **P-3** by exchanging the tosylate ions with triflimide ions. It ought to be noted here that although ionic polymers with the triflimide ion can be made directly from a triflimide-modified monomer **M**, handling the very acidic bistrifluoromethanesulfonyl imide counter ion that would be required is very inconvenient. The chemical structures and purities of **P-4**, **P-5**, and **P-6** ionic polymers were confirmed by elemental, ^1^H, and ^13^C NMR spectra analyses (Figures S2–S4). The facile ring-transmutation polymerization eliminates the need for extreme purification of the monomers or the final polymers. The purification of this class of polymers was conveniently performed by simple dissolution and precipitation in benign solvents, including water. Furthermore, this polymerization reaction requires no special pieces of glassware, no special catalysts, and no rigorous exclusion of moisture, which will enable the scale up for the synthesis of this class of ionic polymers.

Thus, these high-temperature tolerant ionic polymers can represent an attractive alternative to reduce their impact on the environment pollution. The metathesis reaction offers excellent potential for the low-cost mass production of a range of ionic polymers with tunable properties.

### 3.3. Molecular Weight and Solubility

The number-average molecular weights (M_n_s) of the ionic polymers **P-1** to **P-6** ranging from 36 to 65 kDa are summarized in Table 1. Importantly, the polydispersity index (PDI) is low (<1.73) in comparison to other commercially available polymers that have much wider molecular weight distributions. This property provides the polymers with very specific properties that can be tailored for various applications. Based on the molecular weight data of the ionic polymers, the ring- transmutation polymerization reaction appeared to be quite effective for synthesizing phosphorous with high molecular weight containing ionic polymers.

The molecular weight of the polymers can easily be adjusted by utilizing non-stoichiometric ratios of **M** and diamines in the polymerization reaction. In terms of solubility, **P-1**, **P-2**, and **P-3** ionic polymers were found to be readily soluble in methanol, ethanol, and acetonitrile. Polymers **P-4**, **P-5**, and **P-6**, on the other hand, are soluble in acetone and acetonitrile. However, they have no or very poor solubility in water, propanol, toluene, chloroform, tetrahydrofuran, and dichloromethane, suggesting that these polymers have high resistance to water, water vapor, and organic gases, as required for their use as coatings and structural component materials.

### 3.4. Thermal Properties

**Figure 5:**
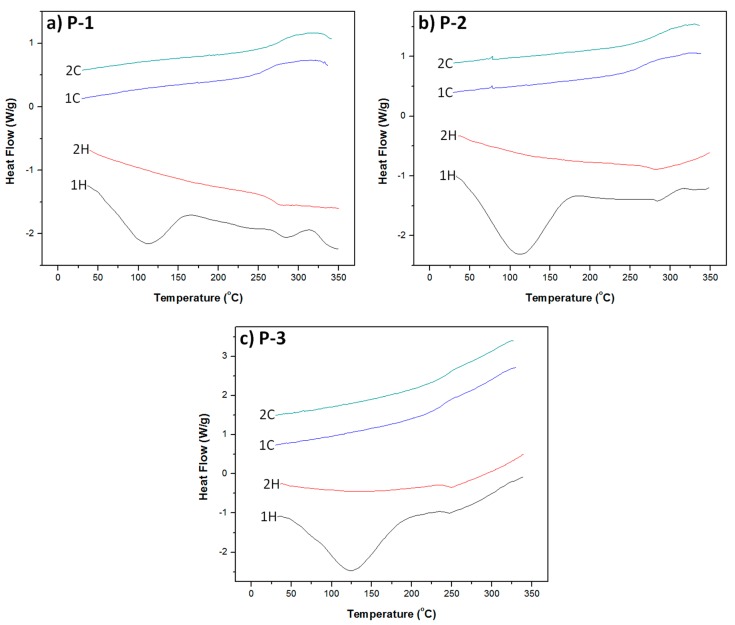
Differential scanning calorimetry (DSC) thermograms of ionic polymers (**a**) **P-1**, (**b**)**P-2**, and (**c**) **P-3** obtained at heating and cooling rates of 10 °C/min in nitrogen.

To determine the thermal properties of the ionic polymers, differential scanning calorimetry (DSC) and thermogravimetric analysis (TGA) were performed at heating and cooling rates of 10 °C/min and at a heating rate of 10 °C/min in nitrogen, respectively. The DSC thermograms of ionic polymers **P-1**, **P-2**, and **P-3** are shown in Figure 5. A broad endotherm at 112 °C due to solvent loss was observed in the first heating cycle of the DSC thermogram of **P-1** (Figure 5a), while no such endotherm was observed in the second heating cycle. The high glass transition temperature of **P-1**—*T*_g_ = 276 °C—was evident in its DSC thermograms. However, with further heating, an exotherm was observed after the *T*_g_ at 320 °C, which was due to cold crystallization. A glass transition temperature of 275 °C was observed for **P-2** (Figure 5b), whereas no cold crystallization endotherm was observed at a higher temperature up to 350 °C. For **P-3**, a glass transition temperature of *T*_g_ = 243 °C was observed (Figure 5c). For triflimide counterions containing ionic polymers **P-4**, **P-5**, and **P-6**, *T*_g_ in the range of 230 to 253 °C was observed by DSC analysis. Depending on the chemical structures of the polymers, the *T*_g_ ranged from 231 to 275 °C (Table 1). These results indicate that these ionic polymers have high *T*_g_ values.

**Figure 6:**
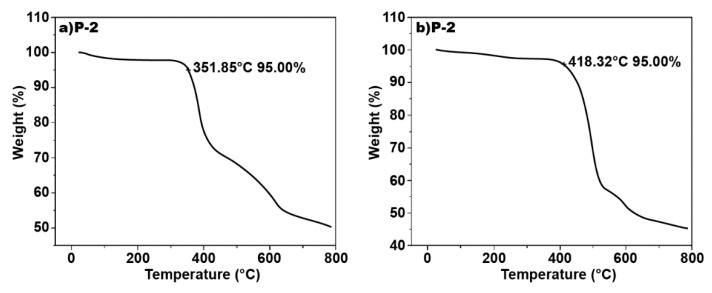
Thermogravimetric analysis (TGA) plots of two representative ionic polymers **P-2** and **P-4** obtained at a heating rate of 10 °C/min in nitrogen.

Figure 6 shows the TGA plots of two representative ionic polymers **P-2** and **P-4** obtained at a heating rate of 10 °C/min in nitrogen. The decomposition temperatures (T_d_s) of the ionic polymers are also compiled in Table 1. The T_d_ values were found to be in the temperature range of 343 to 352 °C for the tosylate containing ionic polymers (**P-1**, **P-2**, and **P-3**), and 418 to 439 °C for the triflimide containing ionic polymers (**P-4**, **P-5**, and **P-6**), at which only a 5% weight loss of all six ionic polymers occurred. These decomposition temperatures were more than 100 °C higher than that of a poly(*p*-phenylene-diphenylpyridinium) ionic polymer (245 °C) that does not contain the phosphine oxide moiety. In general, polymers containing the tosylate counterions have a higher *T*_g_ but a lower T_d_ compared to those containing triflimide counterions due to the high thermal stability of the fluorinated anion. The TGA analyses also revealed that all six ionic polymers (**P-1** to **P-6**) have a high char yield at 700 °C (52% for **P-1**, 53% for **P-2**, 49% for **P-3**, 54% for **P-4**, 47% for **P-5**, and 45% for **P-6**). These results suggest that these ionic polymers have high T_d_ values and char yields.

### 3.5. Processing, Moisture, and Vapor Resistance, Optical/Fluorescence Properties, Thin Films, Adhesion, Mechanical and Tensile Strength

The ionic polymers **P-1**, **P-2**, and **P-3** were found to be readily soluble in methanol, ethanol, and acetonitrile, whereas **P-4**, **P-5**, and **P-6** are soluble in acetone and acetonitrile because of different triflimide counterions. However, these ionic polymers have no or very poor solubility in Water, propanol, toluene, chloroform, tetrahydrofuran, and dichloromethane. The solubility profiles of these ionic polymers are summarized in Table 2. These results indicate that the phosphine oxide-containing ionic polymers have high resistance to moisture, water vapor, and organic gases, which are prerequisite criteria for coatings and structural component materials.

**Figure 7:**
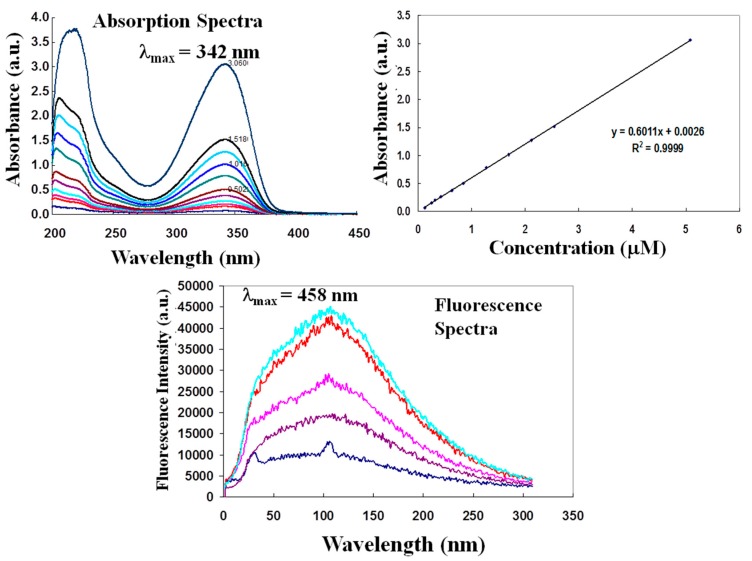
Optical absorption and fluorescence spectra of ionic polymer **P-1** in methanol (concentration = 10^−6^–10^−4^ M).

**Figure 8:**
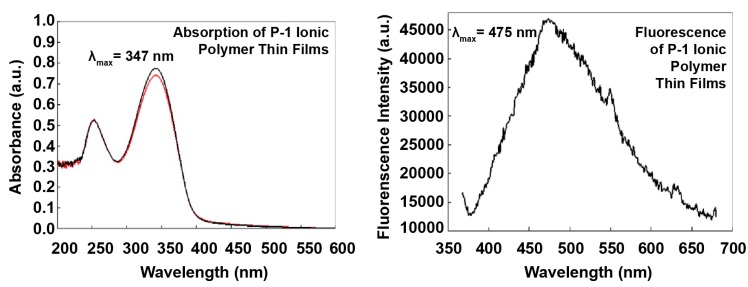
Absorption (**left**) and fluorescence (**right**) spectra of **P-1** thin films on glass substrates.

**Figure 9:**
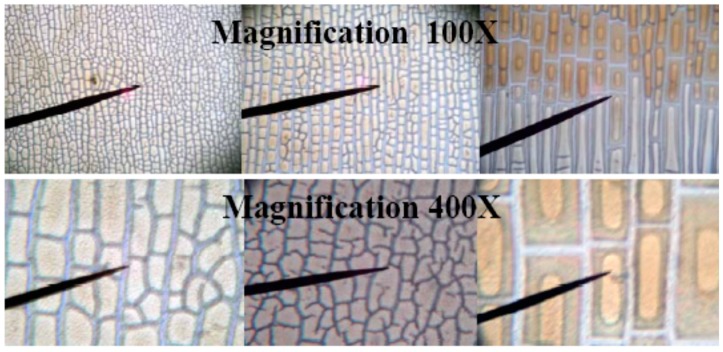
Optical micrographs of thin films of **P-1** on glass substrates.

The optical absorption and fluorescence properties of these ionic polymers were examined using a Shimadzu UV-2401PC UV-Vis spectrophotometer and PTI QuantaMaster™ Model QM-4/2005 spectrofluorometer, respectively. Figure 7 shows the absorption and fluorescence spectra of **P-1** in methanol. The **P-1** polymer absorbs in the UV-visible region (200–400 nm) with absorption maximum (λ_max_) at 342 nm, which is due to π–π^*^ transition. **P-1** has a high molar absorption coefficient (~10^5^ M^−1^cm^−1^) and shows linear dependence with concentration. In methanol solution, the **P-1** polymer emits blue light with a peak maximum at 458 nm. Similar optical absorption and fluorescence properties in methanol solution were also observed for ionic polymers **P-2** to **P-6**. These polymers were found to absorb light in the wavelength range of 200 to 400 nm, and emit light in the UV-visible range (350 to 650 nm). Both absorption and emission intensities increase linearly with the increased polymer concentration between 0.1–5 μM. These ionic polymers have excellent thin-film forming properties. Figure 8 shows the representative absorption and fluorescence spectra of **P-1** polymer thin films coated on glass substrates by spin coating. Thin films of **P-1** to **P-6** also absorb in the 200 to 400-nm regions with peaks at ~245 nm. They exhibit weak fluorescence, as expected. The thin films of the ionic polymers form aligned structures, as observed by optical microscopy (Figure 9). The formation of aligned structures of the thin films of ionic polymers was further examined by atomic force microscope (AFM) analysis, as discussed below. Due to the aligned structure, these ionic polymers can be used for enhancing the performance of optoelectronic devices.

**Figure 10:**
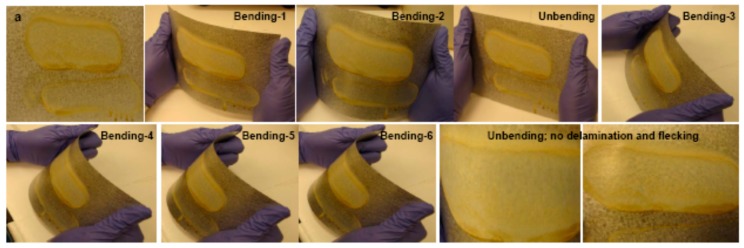
Photographs of bending–unbending of large-area thin films of **P-1** on tin substrates prepared by spray coating from methanol solution.

To understand the adhesion, delamination, mechanical, and tensile strength of the ionic polymers, large-area thin films of the ionic polymers on metal substrates were prepared by spray coating. Figure 10 shows the large-area thin films of the ionic polymer **P-1** on tin metal substrates (without any surface treatment of the metal substrate) prepared by spray coating from methanol solutions. The thin films retained their quality even after bending–unbending the metal substrates at different angles. They also retained the quality of the thin films over a long period of time. No delamination and flecking of the thin films were observed while bending–unbending the metal substrates (Figure 10). Similar properties were also observed for ionic polymers **P-2** to **P-6**. These results suggest that they show excellent adhesion to metal substrates, high mechanical and tensile strength, and have excellent application potential as coating and structural component materials for automobiles, aircrafts, engines, and power/propulsion systems.

### 3.6. High-Temperature Heat Treatment and Morphologies of Thin Films

**Figure 11:**
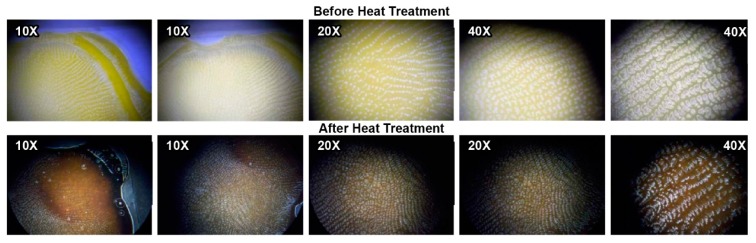
Optical micrographs of thin-films of ionic polymer **P-1** before and after heat treatment at 550 °C in air for 30 min at different magnifications.

The thermal stability of the ionic polymers was also determined by heat treating their thin films at 550 °C in air for 30 min, and probing their morphologies before and after the heat treatment, as shown in Figure 11. After heat treatment at 550 °C in air for 30 min, a change of color of the thin films from yellow to brown was observed, which may be due to the phosphine oxide group forming a glassy phosphate layer [79,80,81,82,83,93,94,95]. However, no significant changes in the structural alignment of thin films for ionic polymers were observed (Figure 11).

**Figure 12:**
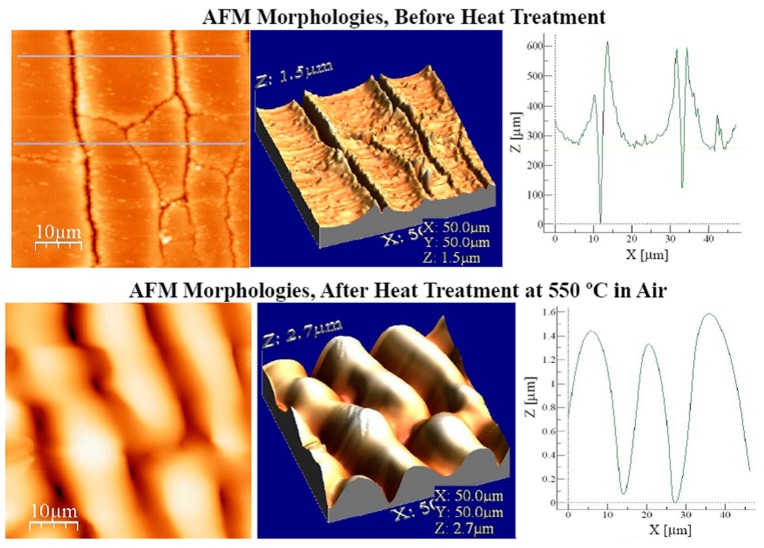
Tapping-mode atomic force microscope (AFM) height, three-dimensional (3D), and cross-section images and data for thin films of ionic polymer **P-1** before and after heat treatment at 550 °C in air for 30 min.

For morphological studies, the thin films of ionic polymers were solvent cast from their methanol solution onto polished silicon substrates (1 × 1 cm), and dried at 60 °C under vacuum for 12 h. Heat-treated samples were prepared by heating the ionic polymer thin films at 550 °C for 30 min utilizing a hot plate. AFM images were obtained using a Nano-Scope III microscope (Digital Instruments Inc., Veeco Metrology group, Santa Barbara, CA) in standard tapping mode with a single silicon-crystal tip as a nanoprobe. The tapping-mode AFM height, 3D, and cross-section images of the surface morphologies of **P-1** ionic polymer thin films on silicon substrates before and after heat treatment are shown in Figure 12. The root mean square (RMS) surface roughness 0.08913/0.61544, average height of domains 0.52786/1.81704, and maximum height of domains 1.07694/3.11783 were observed from the analysis of AFM results obtained from thin films of the **P-1** ionic polymer before/after heat treatment. A higher RMS surface roughness, average height of domains, and maximum height of domains were observed after heat treatment for the **P-1** films compared to that before heat treatment due to the deformation of the surface morphology by the formation of glassy phosphate layers. Similar AFM morphological changes were also observed for the thin films of other ionic polymers **P-2** to **P-6** in the series.

### 3.7. Fire Resistant and Retardant Properties

**Figure 13:**
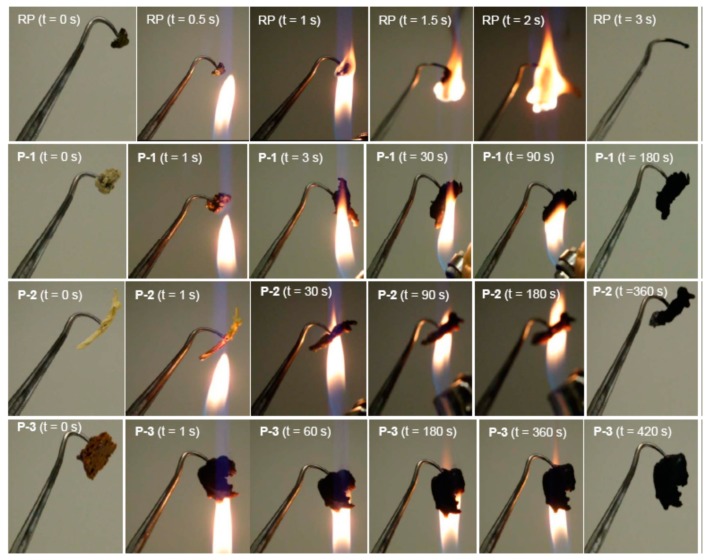
Photographs taken during direct flaming/combustion of a reference polymer (**RP**; top row) and ionic polymers (**P-1**, **P-2**, and **P-3**) (bottom three rows).

**Figure 14:**
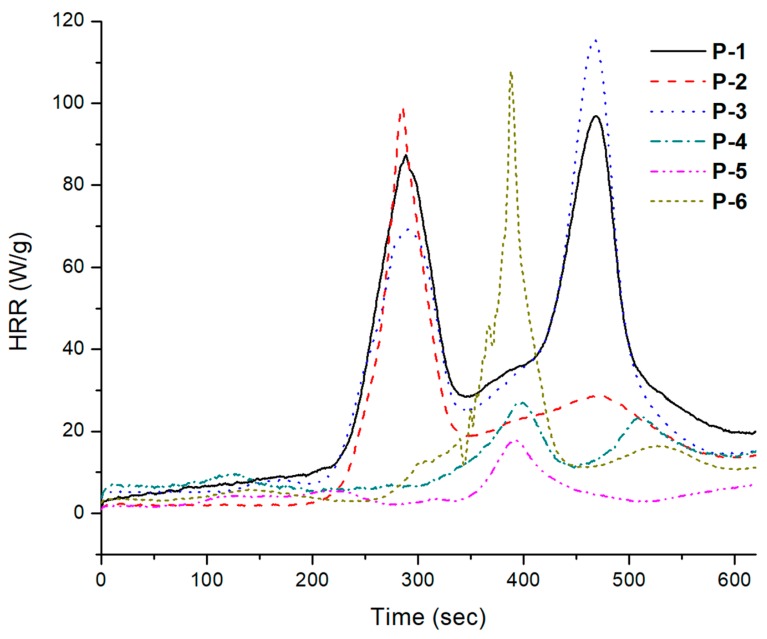
Heat release rate (HRR) of polymers **P-1** to **P-6** vs. time.

The fire resistant and retardant properties of the ionic polymers **P-1** to **P-6** and a reference polymer (**RP**) (polythiophene) were investigated by direct flaming/firing them with a propane torch and probing their burning and/or flame ignition and retardant behavior (Figure 13). A fire test for each of the ionic polymers was a vertical flame test based on the NASA Upward Flame Propagation Test (NASA Standard 6001 and ASTM D6413). All the tests were conducted in a chemical hood utilizing an open design setup in a 20% ambient oxygen environment. All the polymer samples were held firmly by a clean stainless steel hook. A clean piece of paper below the polymer sample was placed for easy viewing of any dripping. Then, the polymer samples were exposed to an ignition source at their bottom edge for 10 s to over 5 min. A propane torch was utilized as an igniter. All the flame studies were videotaped so that the afterflame, afterglow, and char formation times, as well as flame propagation, melting, and dripping could be observed by direct flaming with a propane torch for over 5 min, although deformation and color change from yellow to dark due to char formation were observed (Figure 13). These results indicate that the phenyl phosphine oxide-containing ionic polymers (**P-1** to **P-6**) developed in this study exhibit excellent fire resistant and retardant properties (video clips in Supplementary Materials). Additionally, Figure 14 shows the heat release rate (HRR), in units of W/g of polymers **P-1** to **P-6** over time in seconds as measured by microcalorimetry. It provides useful information about the combustion of the polymers and is effective for the laboratory evaluation of the flame retardant properties of polymers. It measures not only the HRR, but also the peak heat release rate (PHRR); both are considered significant parameters for evaluating the fire retardant properties of materials. Total heat released (THR) is another relevant parameter, which represents the sum of heat released until the flame is extinguished. The data obtained from microcalorimetry for polymers **P-1** to **P-6** are compiled in Table 3, which suggest that this class of ionic polymers are excellent fire retardant polymers, since they exhibit relatively low HRR, PHRR and THR values. Additionally, both **P-4** and **P-5** showed very low PHRR values when compared with **P-6**, which was presumably related to the flexible ether linkages present in **P-6** that facilitate the combustion processes, as opposed to the rigid aromatic moieties present in **P-4** and **P-5**.

### 3.8. Electrochemical Properties

To understand the electronic structures of the ionic polymers **P-1** to **P-6** in relation to the charge transport processes in optoelectronic devices, we performed cyclic voltammetry (CV) measurements on the films of the ionic polymers. The CV measurements were performed on an EG&G Princeton Applied Research potentiostat/galvanostat instrument (model 263A) in an electrolyte solution of 0.1 M of tetrabutylammonium hexafluorophosphate (TBAPF_6_) in acetonitrile. Platinum (Pt) wire electrodes were used as both counter and working electrodes, and the Ag/Ag+ electrode was used as the reference. A ferrocene/ferrocenium (Fc/Fc^+^) redox couple was used as an internal standard. The potential values obtained in reference to the Ag/Ag^+^ electrode were converted to the saturated calomel electrode (SCE) scale. Films of ionic polymers **P-1** to **P-6** were coated on the Pt working electrode by dipping the Pt wire into 0.5–1 wt% methanol solutions, and dried under vacuum at 80 °C for 6 h. All the ionic polymers showed reversible reductions with onset reduction potentials of –1.83 to –1.80 V versus SCE. The formal reduction potentials were also very similar, being in the range of –1.95 to –1.91 V versus SCE. We estimated the electron affinity (EA, LUMO level) of **P-1** to **P-6** to be virtually identical, 2.57–2.59 eV. Irreversible oxidation was observed for all six ionic polymers. By rough estimation, the ionization potential (IP, HOMO level) for these ionic polymers was found to be ca. 5.40 eV. These results suggest that the electrochemical properties of these ionic polymers are determined primarily by the *bis*(2,6-diphenylpyridinium) backbone structure. The observed reversible reduction, high electron affinity, and irreversible oxidation suggest that these ionic polymers are intrinsic n-type (electron transport) and hole-blocking materials.

## 4. Conclusions

Our study focused on synthesizing and characterizing six new phosphine oxide-containing ionic polymers with tunable properties via ring-transmutation polymerization and metathesis reactions. The development of these materials aimed to meet the need for safe, easily processed, tunable materials. The approach followed enables the production of ionic polymers through a simple polymerization reaction with high yields and purity utilizing DMSO as solvent for the polymerization reaction and methanol-water for their purification. This process also permitted the easy adjustment of properties by a simple step such as counterion exchange. Polymers with high glass transition temperatures (*T*_g_ > 230 °C) and relatively high decomposition temperatures greater than 340 °C were achieved. The polymers were found to avoid ignition even after 5 min of exposure to direct fire, demonstrating their ability to act as high-temperature, fire retardant materials. Additionally, microcalorimetry measurements showed that they had relatively low HRR, PHRR, and THR values, which suggested that this class of ionic polymers had excellent fire retardant properties. These polymers were readily resistant to moisture and common organic solvents, and were found to have excellent film-forming properties. We also demonstrated the ability of these polymers to absorb light in the UV range of the spectrum and produce photoluminescence in the visible region. Photoactive, electroactive, and robust, high-temperature tolerant ionic polymers have high application potentials in the areas of electronics, optoelectronics, fire and corrosion resistant coatings, and structural components for automobiles, aircraft, engines, power, and propulsion systems. In addition, they can be used for firefighter garments, printed circuit boards, construction materials, and paper-thin coatings for protecting bonds, securities, stock certificates, real estate titles, and deeds.

## Figures and Tables

**Table 1:** Thermal properties and gel permeation chromatography (GPC) data of ionic polymers **P-1** to **P-6**.

Polymer	*T*_g_ (°C)	*T*_d_^a^ (°C)	Char Yield at 700 °C (%)	GPC Results	
				M_n_ (Da)	PDI
P-1	270	348	52	36,559	1.49
P-2	275	352	53	51,261	1.29
P-3	258	343	49	64,545	1.31
P-4	253	439	54	43,933	1.73
P-5	231	418	47	53,783	1.50
P-6	243	437	45	64,264	1.42

^a^*T*_d_ is the decomposition temperature at which the mass of the polymer was reduced by 5 wt% of the original.

**Table 2:** Solubility profiles of ionic polymers **P-1** to **P-6** in different solvents.

Solvent	P-1	P-2	P-3	P-4	P-5	P-6
H_2_O	+ –	–	–	–	–	–
MeOH	+	+	+	–	–	–
EtOH	+	+	+	–	–	–
*n*-Propanol	+ –	–	+ –	+ –	–	–
Acetone	+ –	+ –	+ –	+	+	+
Toluene	–	–	–	–	–	–
CHCl_3_	–	+ –	+ –	–	+ –	–
THF	–	+ –	+ –	–	+ –	–
CH_2_Cl_2_	–	+ –	+ –	–	–	–
CH_3_CN	+	+	+	+	+	+

+ = Soluble, + – = Slightly soluble, – = Insoluble.

**Table 3:** Microcalorimetry data of polymers **P-1** to **P-6**.

Sample	HRR (J/g.K)	Peak HRR (W/g)	THR (kJ/g)	T_PHRR_ (°C)	Char (%)
P-1	81	75.83	11.3	392.9	51.45
	80	77.87	10.8	391.8	52.17
	85	84.41	10.4	393.8	51.53
P-2	93	92.8	7.1	389.1	51.91
	90	89.88	7.5	390.3	51.19
	76	75.69	7.3	394.6	52.31
P-3	103	103.4	12.0	579.5	50.99
	93	93.7	11.8	581.8	51.61
	103	103.2	12.5	565.8	51.09
P-4		19.9			55.76
		21.3			57.07
		20.7			53.87
P-5		16.3			48.63
		14.5			48.07
		16.5			47.27
P-6	98	99.67	3.9	488.5	45.64
	71	71.79	3.6	485.5	46.73
	92	92.64	3.6	483.5	45.28

## Supplementary Materials

The following are available online at https://www.mdpi.com/2073-4360/11/7/1141/s1, Figures S1–S4: ^1^H and ^13^C NMR spectra of polymers **P-3**, **P-4**, **P-5** and **P-6**, Video: polymers **P-1**, **P-2**, **P-3**, **P-4** and reference polythiophene.
