# Supplementary material for: Phosphine Oxide Containing Poly(pyridinium salt)s as Fire Retardant Materials
Source: Polymers (Basel). 2019 Jul 3;11(7):1141. doi: 10.3390/polym11071141 (PMC6680757; doi:10.3390/polym11071141)
Supplement: Supplementary file 1 [file polymers-11-01141-s001.zip › Alam et al - supplementary materials.docx]

**Phosphine Oxide Containing Poly(pyridinium salt)s as Fire Retardant Materials**

**Maksudul M. Alam,^1^ Bidyut Biswas,^2^ Alexi K. Nedeltchev,^2^ Haesook Han,^2^ Asanga D. Ranasinghe,^2^ Pradip K. Bhowmik,^2^* Kisholoy Goswami^1^**

^1^InnoSense LLC, 2531 West 237th Street, Torrance, California 90505, USA

*^2^Department of Chemistry and Biochemistry, University of Nevada Las Vegas, 4505 Maryland Parkway Box 454003, Las Vegas, Nevada 89154-4003, USA*

**Figure S1.** ^1^H and ^13^C NMR spectra of **P-3** in *d_6_-*DMSO taken at room temperature.

**Figure S2.** ^1^H and ^13^C NMR spectra of **P-4** in *d_6_-*DMSO taken at room temperature.

**Figure S3.** ^1^H and ^13^C NMR spectra of **P-5** in *d_6_-*DMSO taken at room temperature.

**Figure S4.** ^1^H and ^13^C NMR spectra of **P-6** in *d_6_-*DMSO taken at room temperature.

Five video clips for demonstrating fire retardant properties of poly(pyridinium salt)s:
